# The COL-4A1 polypeptide destroy endothelial cells through the TGF-β/PI3K/AKT pathway

**DOI:** 10.1038/s41598-021-94801-5

**Published:** 2021-08-03

**Authors:** Ting Li, Zhonghui Ling, Kaipeng Xie, Yixiao Wang, Zhijing Miao, Xiaohong Ji, Jingyun Li, Wenwen Hou, Qiuqin Tang, Xiaojie Yuan, Nan Li, Chanjuan Li, Hongjuan Ding

**Affiliations:** Women’s Hospital of Nanjing Medical University, Nanjing Maternity and Child Health Care Hospital, The Affiliated Obstetrics and Gynecology Hospital of Nanjing Medical University, Nanjing, China

**Keywords:** Cell biology, Genetics, Molecular biology, Medical research, Molecular medicine, Pathogenesis

## Abstract

Preeclampsia (PE) is commonly considered as a placental disorder in pregnancy. Until now, the etiology and pathological mechanism of PE have remained ambiguous. Although PE can lead to a variety of maternal and infant complications, there are still no effective treatments. This study aimed to explore the correlation between the novel polypeptide COL-4A1 and PE, and to identify the underlying mechanism by which this polypeptide may function and to explore new therapeutic targets for PE. A rat model of PE was established and used to verify the function of the polypeptide COL-4A1 in vivo. Additionally, human umbilical vascular endothelial cells (HUVECs) were cultured with or without COL-4A1 and TNF-α (20 ng/ml). Cell Counting Kit-8 (CCK-8), wound-healing, Transwell and tube formation assays were used to evaluate cell proliferation, migration and angiopoiesis. RNA sequencing and mass spectrometry were conducted to explore the underlying downstream mechanism of COL-4A1. In vivo, COL-4A1 increased blood pressure and elevated the risk of fetal growth restriction (FGR) which was induced by lipopolysaccharide (LPS) in the rat model. In vitro, COL-4A1 significantly inhibited the proliferation and migration of HUVECs. After culture with COL-4A1, compared to control group the adhesive ability and level of reactive oxygen species (ROS) were enhanced and tube formation ability was decreased. Furthermore, Western blotting (WB) and pull-down assays were conducted to explore the underlying mechanism by which COL-4A1 functions, and the TGF-β/PI3K/AKT pathway was identified as the potential pathway involved in its effects. In summary, these results revealed that the polypeptide COL-4A1 caused PE-like symptoms in cells and a rat model. Through the TGF-β/PI3K/AKT pathway, COL-4A1 interferes with the pathogenesis of PE. Thus COL-4A1 is expected to become a potential target of PE, providing a basis for exploring the treatment of PE.

## Introduction

Preeclampsia (PE), a common complication and multiorgan disorder during pregnancy, is commonly considered as a placental disorder and affects 5–8% of pregnant women^1,2^. After 20 weeks gestation, women with PE experience hypertension with or without proteinuria and renal, liver or neurological organ dysfunction, or fetuses experience with fetal growth restriction (FGR)^3^. PE is also one of the highest causes of morbidity and mortality in pregnant women and leads to a variety of short-term and long-term complications in mothers and infants, including cardiovascular diseases, renal disease, metabolic disorders, placental abruption, FGR, and preterm birth^3–8^. Early prediction of PE is still lacking, and diagnosis is mainly based on clinical symptoms. However, although antihypertensive drugs and therapeutic labor induction can alleviate the symptoms of PE, there are still no effective treatments for PE. Therefore, to explore the mechanism and target of PE, and to search for a new effective treatment has always been a research topic in this field^9^.

Until now, the etiology and pathological mechanism of PE have remained ambiguous. Studies have shown that genetics, oxidative stress, inflammation, immune and environmental factors are related to the PE, which has been described with the two-stage placental model theory^10–14^. Numerous reports have suggested that placental factors are the main factors in the pathogenesis of PE^3,13–15^. For example, disorder of trophoblast cell migration and invasion resulted in shallow placental implantation. Insufficient uterine spiral artery remodelling led to narrow vessels, placental ischaemia, hypoxia and vascular endothelial damage^14^. At the same time, some inflammatory factors and antiangiogenic factors, such as tumour necrosis factor α (TNF-α) and endothelin-1 (ET-1), vascular endothelial growth factor (VEGF), placental growth factor (PIGF), and soluble fms-like tyrosine kinase-1 (sFLT-1), are released into the maternal blood circulation, which ultimately caused the mother developing PE^14,16–19^.

Recently, polypeptides have become a research focus in therapeutics. Information regarding the potential effects of peptides on PE remains ambiguous and scant. With the development of polypeptide omics and progress in mass spectrometry, our team screened serum polypeptides from healthy controls and PE patients through liquid chromatography-tandem mass spectrometry (LC–MS/MS), and found that the collagen alpha-1 (IV) chain was significantly up-regulated in patients with PE^20^. We named this polypeptide COL-4A1. There have been some reports about the precursor proteins of COL-4A1. In 1997, collagen IV was firstly found to be related to the proliferation and differentiation of cells in the maternal stroma^21^. In 2003, a study indicated that collagen IV is associated with the migration of trophoblast cells in bovine placentomes^22^. Some studies have shown that collagen IV is related to angiogenesis and cell proliferation^23,24^. In 2018, a review summarized the increasing evidence indicating the role of the collagen alpha-1 (IV) chain in cardiovascular disease (CVD), and proteomic analysis revealed increased collagen type IV in the nonatherosclerotic arteries of patients with type 2 diabetes, but the mechanism is unknown^25,26^. Although, there are some studies on precursor proteins of COL-4A1, the function of the polypeptide is still unclear. Moreover, researches on the function and underlying mechanisms of the polypeptide COL-4A1 in PE remain scant. In the current study, we used an animal model and cell culture to explore the correlation between the novel polypeptide COL-4A1 and PE. And to uncover its underlying mechanism for the development of new therapeutic strategies for PE.

## Material and methods

### The peptide

The biological macromolecule of COL-4A1 polypeptide consists of a 7-amino-acid sequence (ILGHVPG) and its molecular weight is 2562.02 kDa. Because of the low lipophilicity and weak alkalinity of COL-4A1, we linked a cell-penetrating peptide derived from the HIVTAT sequence (Ac-GRKKRRQRRRPPQQ, Biotin-GRKKRRQRRRPPQQ: for pull-down) to its N terminus. The COL-4A1 polypeptide we used in the present study was synthesized by Shanghai Science Peptide Biological Technology Co., Ltd., and its purity was > 95%. The COL-4A1 polypeptide was dissolved in water to obtain the diluted peptide at different concentrations.

### Animals experimental protocol

All animal experiments were conducted following the guidelines of the Institutional Animal Care Committee and experiments were carried out in compliance with the ARRIVE guidelines. The animal experimental protocols were approved by the Nanjing Medical University Animal Ethics Committee (Approval approval No.: 2005040). Sprague–Dawley (SD) rats (10–12 weeks old, 200–250 g) were purchased from the Experimental Animal Center of Viton Lihua Co., Ltd. According to previous studies, the injection of LPS (Sigma, USA) into rats can cause PE-like symptoms, forming a PE rat model^27,28^. The rats were raised in a light-, humidity- and temperature-controlled environment with free access to food and water under specific pathogen-free (SPF) conditions at the Department of Nanjing Yutong Biotechnology Co., Ltd. After 1 week of acclimatization, vaginal smears for sperm were performed in the morning after females had been housed overnight with a fertile male at a 2:1 ratio. Positive vaginal smears confirmed pregnancy and were used to designate day 0 of pregnancy (gestational day 0, GD 0). On GD 0, pregnant rats were randomly separated into 3 groups: group 1 (saline) (n = 6), group 2 (PE) (n = 6) and group 3 (PE + the COL-4A1 polypeptide) (n = 6). On GD 5 and GD 7, group 1 rats received saline (1.0 mg/kg) via the tail vein, group 2 rats were treated with lipopolysaccharide (LPS) (1.0 μg/kg) (Sigma Aldrich, USA) via the tail vein, and group 3 (PE + the COL-4A1 polypeptide) rats were pretreated with LPS (1.0 μg/kg) via the tail vein. Then, the rats in group 3 were intraperitoneally (ip) administered COL-4A1 (5 mg/kg) on GD 11, GD 14 and GD 17. On GD 20, the rats were euthanized; blood samples were obtained from the inferior cava vena. Serum was stored at − 80 °C for further assessment. Fetal rats and placenta and kidney tissues were collected and further analyzed.

### Blood pressure and urinary protein levels

Systolic blood pressure (SBP) was monitored with a Non-invasive Volume Pressure Recording (VPR) Blood Pressure-monitoring Tail-cuff Plethysmograph (Softron, Japan) on GD 4, 7, 11, 15 and 19^28^. Ten measurements were taken from each rat at a time. On GD 19, 24-h urine samples were collected from the rats and rats were fasted but free to drink water in standard rat metabolic cages. Urine samples were centrifuged at 2000 rpm for 15 min at room temperature, and supernatants were stored at − 80 °C for subsequent analysis. Urine protein and urine creatinine levels were measured using a urine protein quantitation kit (C035-1, Nanjing, China) and creatinine assay kit (C011-2-1, Nanjing, China), respectively.

### Cell culture

HUVECs were purchased from American Type Culture Collection (ATCC, USA). They were cultured in DMEM (GIBCO, USA) with 10% fetal bovine serum (FBS) (GIBCO, USA) and 100 U/ml penicillin and 100 µg/ml streptomycin (GIBCO). HUVECs were used for experiments when cultured in the third to sixth generation. The cells were maintained in a humidified incubator at 37 °C with 5% CO_2_ and 95% O_2_.

### Cell counting Kit-8 assay (CCK-8) for cell proliferation

HUVECs cells were seeded into 96-well plates at 3 × 10^3^ per well. Cells were treated with or without COL-4A1 and TNF-α (20 ng/ml). A CCK-8 cytotoxicity assay (Dojindo, CK04-13, Japan) was used to evaluate the proliferative capacity of the HUVECs. After the cells had attached, according to the manufacturer’s instructions, we added 10 µl of CCK-8 solution to the medium and then incubated the cells at 37 °C and 5% CO_2_ for 2 h. Finally, a multifunctional microplate reader was used to record the cell OD value at 450 nm at 0 h (Hybrid Technology, BioTek, USA). Additionally, we recorded the cell OD values at 24 h, 48 h and 72 h. The experiments were conducted with triplicate samples and repeated three times.

### Wound-healing assay and Transwell assay of migration

HUVECs migration was assessed by wound-healing and Transwell assays, and the differences among cells treated with different concentrations of COL-4A1 (0 μM, 30 μM) were compared. Wound-healing assay: When the cells were full covered in a six-well plate, a 100-µl tip was used to scratch a straight line through the middle of each well. Then, we used PBS (GIBCO, USA) washing to remove necrotic cells 3 times, after which the cells were cultured in serum-free DMEM; this time point was set as 0 h. We photographed the scratch width per well at 0 h and 48 h. The following ratio was used to reveal the wound healing rate: (scratched area at 0 h − scratched area at 48 h)/scratched area at 0 h. Transwell assay: In the upper chambers of the wells of a Transwell plate (3428, Corning, USA), a total of 5 × 10^4^ cells were seeded with 200 μl of serum-free DMEM. To the lower chambers, we added 700 μl of DMEM containing 20% FBS. After 36 h of incubation at 37 °C with 5% CO_2_, we processed the Transwell chamber by the addition of 700 μl of paraformaldehyde (4%) at 4 °C for 30 min to fix the cells and 700 μl of 1% crystal violet to stain the cells for 30 min in the dark, after which noninvaded cells in the upper chamber were removed with a cotton swab. We randomly selected and photographed three different fields, and images were captured at 100× magnification with a ZEISS inverted microscope (ZEISS, Germany). The migrated cells were counted, and the average value was recorded. The experiments were repeated three times.

### In vitro tube formation assay

HUVECs were cultured with different concentrations of COL-4A1 (0 μM, 30 μM) and TNFα (20 ng/ml) at 37 °C and 5% CO_2_ for 24 h. A 48-well cell culture plate was coated with 100 μl/well undiluted Matrigel basement membrane matrix (Corning 356234, USA) and incubated at 37 °C for 30 min to allow the Matrigel to coagulate. Cells in the control group and those treated with COL-4A1 (30 μM) as described above were collected. The HUVECs were counted with a counting chamber (Life Technology, USA), and 8 × 10^4^ cells per well were seeded on wells coated with Matrigel^28^. Three replicate wells were set per group, and the cells were incubated for 16–20 h at 37 °C under 5% CO_2_ to allow tube formation. The angiogenesis status was assessed, and images were captured at 100× magnification using a ZEISS inverted microscope (ZEISS, Germany). The experiments were repeated three times.

### Reactive oxygen species (ROS) assay

A Reactive Oxygen Species Assay Kit (ROS Assay Kit, Yeasen, China) was used with 2,7-dichlorofluorescin-diacetate (DCFH-DA) to detect intracellular ROS levels. Cells were incubated with an appropriate volume of 10 μM DCFH-DA diluted in serum-free DMEM at 1:1000 for 30 min at 37 °C in the dark. Then, serum-free DMEM was used to wash the cells three times to remove the DCFH-DA that had not entered the cells. Finally, the samples were directly observed and photographed at 100× magnification with a laser confocal microscope (ZEISS, Germany). A fluorescence microplate reader (Hybrid Technology, BioTek, USA) was used to detect the fluorescence intensity of each sample; the excitation wavelength was 488 nm, and the emission wavelength was 525 nm. All samples were analyzed in triplicate, and the experiment was repeated three times.

### Monocyte adhesion assay

HUVECs were seeded in 96-well plates at 1 × 10^4^/well and pretreated with or without COL-4A1 and TNF-α (20 ng/ml) for 24 h. According to the manufacturer's instructions, THP-1 cells (1 × 10^6^ cells/ml) were stained with calcein-AM (AAT BioQuest, USA) for 1 h at 37 °C with 5% CO_2_. Then, the stained THP-1 monocytes (200 μl/well) were added to co-incubate with HUVECs for 1 h at 37 °C with 5% CO_2_ and washed three times with PBS to wash away the unadhered THP-1 monocytes. Images were captured at 100× magnification with a ZEISS fluorescence microscope (ZEISS, Germany). The assay was repeated three times.

### RNA isolation and quantitative reverse transcription polymerase chain reaction (qRT-PCR)

TRIzol (700 µl) reagent (Invitrogen Inc., Carlsbad, CA, USA) was used to extract total RNA from samples, and a RevertAid First Strand cDNA Synthesis Kit (Thermo, USA) was used to synthesize cDNA. The concentration and purity of the extracted RNA and cDNA were detected using NanoDrop technology (Agilent, Santa Clara, CA, USA). According to the manufacturer's instructions, cDNA, ddH_2_0, primers (TaqMan Gene Expression Assays, Applied Biosystems) and SYBR Green (Thermo, USA) were added to 384-well plates and further processed in a ViiA7 Real-time PCR System from Life Technologies (Applied Biosystems, USA). All primers used for amplification are shown in Table 1. GAPDH was used to normalize all gene expression results. The analysis of each qRT-PCR was performed in triplicate. gene expression fold changes were calculated using the 2^−∆Ct^ method^29^.

**Table 1:** Primer sequence for qRT-PCR.

Gene name	Forward primer (5′–3′)	Reverse primer (5′–3′)
VEGF	AGGTCCCCTTCTTCAGGAAACG	TCCAGGCTGTGCTCAGGAAAAG
PIGF	GAGACCCACAGACTGCCAC	ACCTTGGCCGGAAAGAACAA
sFLT-1	TTTGCCTGAAATGGTGAGTAAGG	TGGTTTGCTTGAGCTGTGTTC
ICAM-1	CAATGTGCTATTCAAACTGCCC	CAGCGTAGGGTAAGGTTCTTG
VCAM-1	CAAAGGCAGAGTACGCAAAC	ACAGGATTTTCGGAGCAGG
GAPDH	GGAGCGAGATCCCTCCAAAAT	GGCTGTTGTCATACTTCTCATGG

### RNA sequencing

We isolated total RNA from HUVECs in 6-well plates treated with COL-4A1 (0 μM, 30 μM) for 24 h with three replicates per group. The purity was assessed and RNA single-cell sequencing of each sample was conducted with the free online Majorbio Cloud Platform (Shanghai Majorbio Bio-pharm Technology Co., Ltd). We also performed Gene Ontology (GO) and Kyoto Encyclopaedia of Genes and Genomes (KEGG) pathway analysis (KOBAS, Version 2.1.1, http://kobas.cbi.pku.edu.cn/download.php). Additionally, a Venn diagram, volcano plot and heatmap were prepared (edgeR, Version 0.46.0, http://bioconductor.org/packages/stats/bioc/edgeR/).

### Pull-down assays and mass spectrometry assays

According to the instructions, we firstly took out 30 µl beads and put them into a 5 ml centrifuge tube (Invitrogen, 00875123). Firstly, we used the lysate (RIPA buffer, Beyotime Institute of Biotechnology, China) to wash the beads for 3 times. Then biotin-COL-4A1 (0 μM, 30 μM) were added to the 5 ml centrifuge tube and incubated with beads overnight at 4 °C. Then, the beads were washed another 6 times the protein sample was added and mixed and the beads were incubated overnight at 4 °C. The polypeptide-protein mixture was mixed with loading buffer and subjected to WB to obtain protein expression data. Finally, we performed silver staining to obtain the differential protein fragments and sent them to a company for mass spectrometry assays (BGI, China).

### Western blotting (WB)

WB was used to quantify protein levels in the samples. HUVECs were treated with COL-4A1 (0 μM, 30 μM) for 24 h. Proteins were extracted using RIPA buffer, PMSF (Beyotime Institute of Biotechnology, China) and phosphatase inhibitors (Servicebio, Wuhan). The protein samples were then separated by 10% SDS-PAGE (Servicebio, Wuhan) and transferred to nitrocellulose membranes. Then, the membranes were blocked with 5% skim milk (Guangzhou Saiguo Biotech Co., LTD) for 2 h and incubated with primary antibodies against TGF-β (Abcam, 1:1000, ab179695), PI3K (Abcam, 1:500, ab32569), AKT (CST, 1:1000, #9272), p-AKT (CST, 1:1000, #9271), and β-actin (Proteintech, 1:1000, 66009-1-lg) overnight at 4 °C. On the following day, the membranes were washed 3 times for 10 min/wash and incubated with secondary antibodies (1:5000). Finally, the membranes were washed 3 times for 10 min/wash and exposed to FluorChem M from ProteinSimple (GeneTech Biotechnology, Shanghai).

### Statistical analysis

The in vitro culture related studies were obtained from at least three independent experiments and are presented as the means ± standard deviations (SDs). Statistical analysis and image analysis were performed using GraphPad Prism 7.0 software (GraphPad Software, San Diego, CA, USA), ImageJ and SPSS version 22. Differences between two groups were analyzed by Student’s t-test. *P* < 0.05 was used to indicate statistical significance.

## Results

### COL-4A1 increased blood pressure and the risk of FGR and other PE-like symptoms of in a rat model

**Figure 1:**
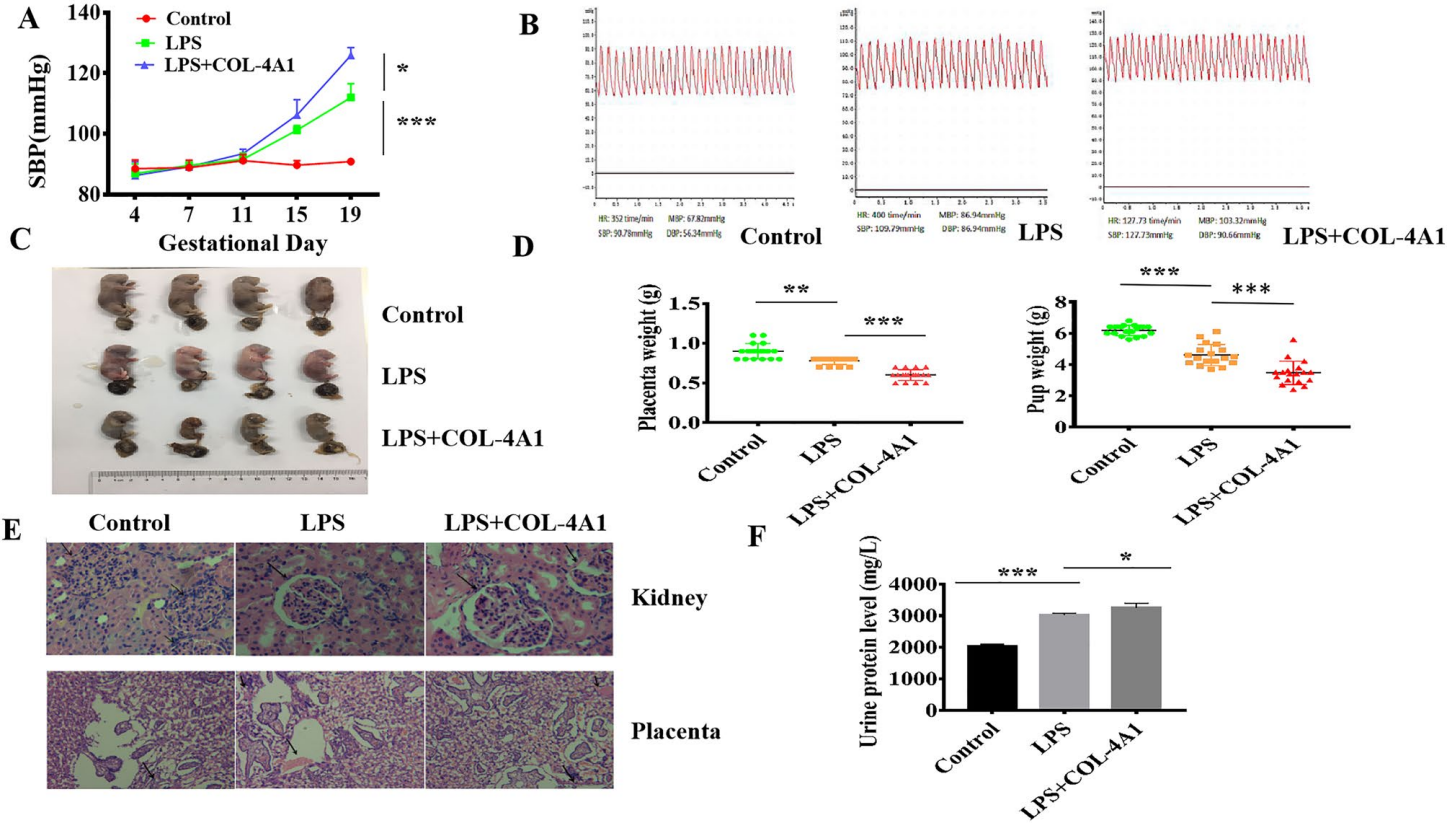
COL-4A1 aggravated PE-like symptoms in a rat model treated with or without LPS. (**A**) The SBP of the control (saline) (n = 6), LPS (PE) (n = 6) and LPS + COL-4A1 (PE + COL-4A1) (n = 6) groups was evaluated on GD 4, 7, 11, 15, and 19. (**B**) Graph showing BP fluctuations in the model rats. (**C**, **D**) Images of the placenta and pup and their weights in each group. (**E**) Kidney and placenta sections from each group were subjected to HE staining. (**F**) Urinary albumin levels on GD 19. *LPS* lipopolysaccharide, *SBP* systolic blood pressure, *GD* gestational day, *PE* preeclampsia. (*P < 0.05, **P < 0.01, ***P < 0.001).

According to the LPS-induced PE-like symptoms, we established an in vivo rat model. As shown in Fig. 1A,B, we observed that the systolic blood pressure (SBP) of the control group (saline, group 1) was essentially unchanged (88.9–90.0 mmHg). In rats that were injected with LPS (group 2), the SBP was significantly increased from GD 11 (*P* < 0.001). In group 3 (LPS + COL-4A1), SBP was also elevated from GD 11, and after GD 15, the difference in SBP was significant (109.4 ± 0.4 mmHg group 2 *vs* 125.9 ± 1.8 mmHg group 3, *P* < 0.05). As shown in Fig. 1C,D, the placental and pup weights of groups 2 and 3 were decreased compared with those of group 1 (All *P* < 0.001). Compared to those of group 2, the placental (0.78 ± 0.01_group 2_ g *vs* 0.60 ± 0.02_group 3_ g, *P* < 0.001) and pup weights (4.61 ± 0.16_group 2_ g *vs* 3.46 ± 0.17_group 3_ g, *P* < 0.001) of group 3 were further decreased. PE can cause pathological changes in the kidney and placenta, and our results were consistent with this finding. Compared to the control group, both group 2 and group 3 showed oedema and inflammatory changes in the kidney and inflammatory cell infiltration and fibrin deposition in the placenta (Fig. 1E). Through analysis of the rat urine, we obtained urine proteins and found their levels to be elevated in groups 2 and 3 (*P*_LPS_ < 0.001, *P*_LPS+COL-4A1_ < 0.05, Fig. 1F).

### The proliferation and migration of HUVECs were significantly inhibited by COL-4A1

**Figure 2:**
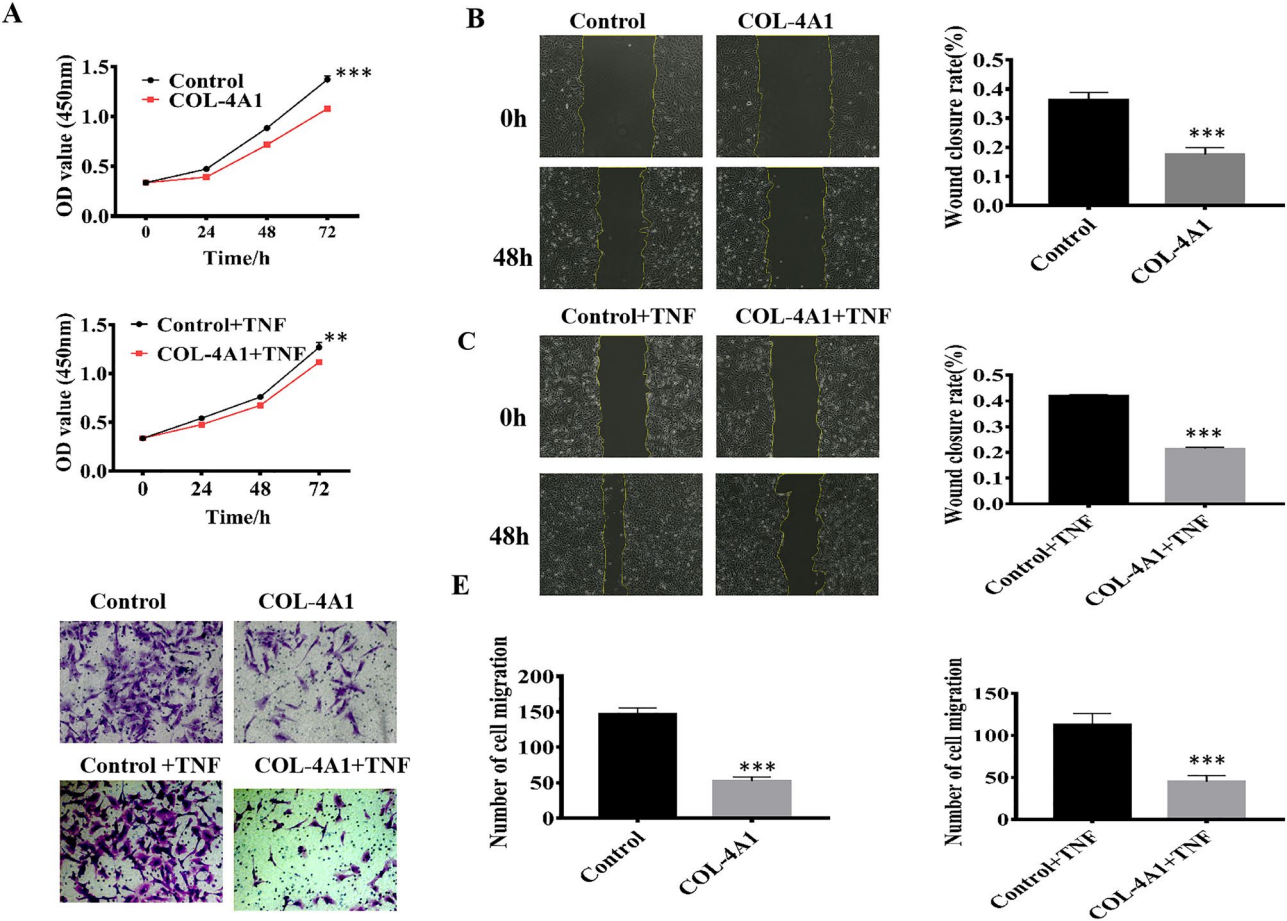
The proliferation and migration of HUVECs were significantly inhibited by COL-4A1. (**A**) HUVECs were treated with or without COL-4A1 and with or without TNF-α before CCK-8 proliferation assays. (**B**–**E**) Wound-healing and Transwell assays were used to detect the ability of in the cells in each group to migrate. All images were captured at ×200 magnification. (*P < 0.05, **P < 0.01, ***P < 0.001).

Based on our *vivo* PE-like rat model results, we further evaluated proliferation and migration using an in vitro cell model. CCK-8 assays were used to measure the proliferation ability. As Fig. 2A shows, regardless of whether TNF-α had been added, the results were consistent and showed that COL-4A1 inhibited proliferation (*P* < 0.001, *P* < 0.01). Wound-healing and Transwell assays were performed to detect the ability of the HUVECs to migrate. Compared to the control and control + TNF-α groups, in the COL-4A1 and COL-4A1 + TNF-α groups the ability of migrate in the wound-healing and Transwell assays were inhibited (all *P* < 0.001, Fig. 2B–E).

### Effects of COL-4A1 on tube formation, the levels of ROS, and adhesion

**Figure 3:**
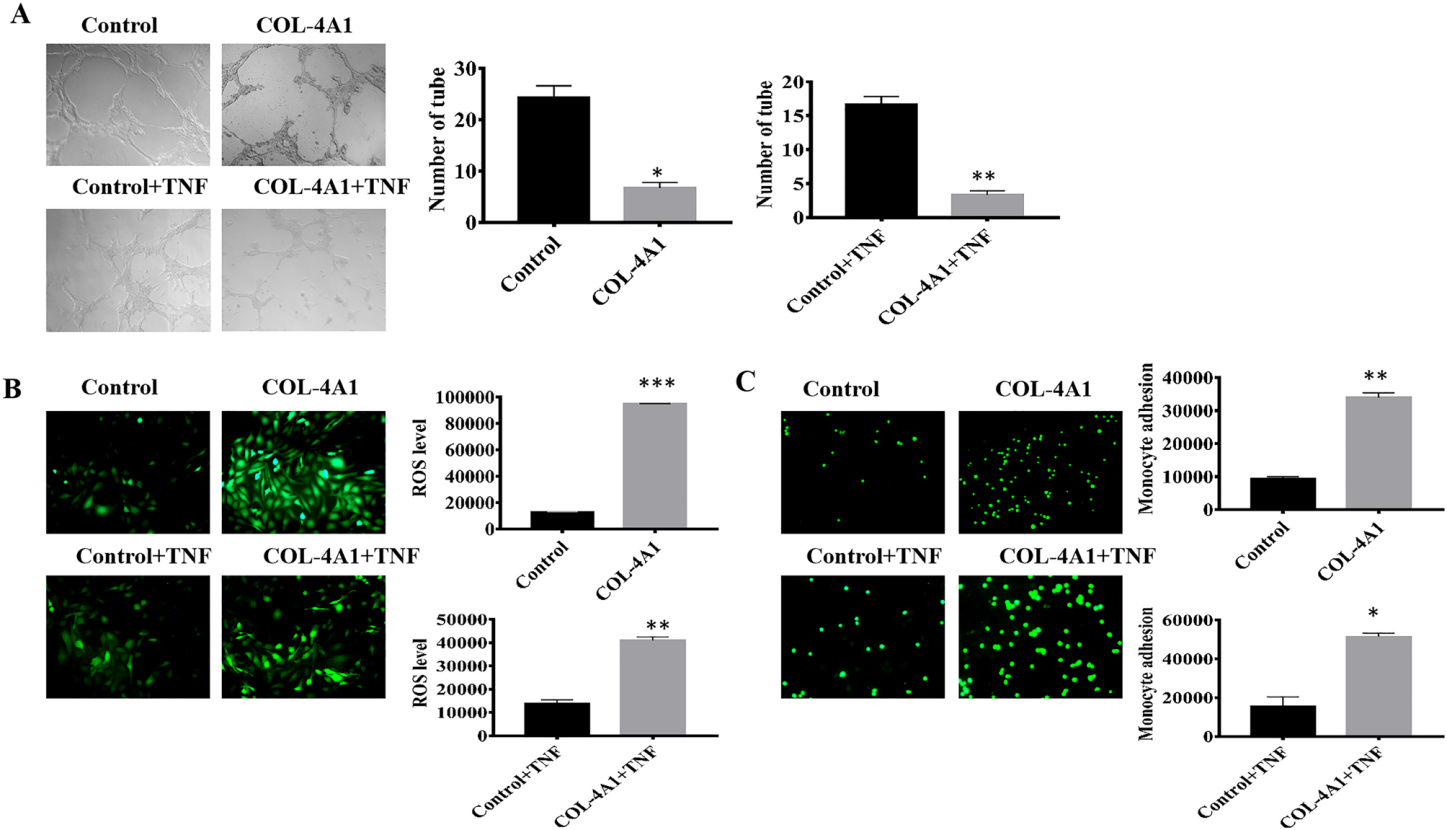
Effects of COL-4A1 on tube formation, ROS levels, and adhesive ability. (**A**) Qualitative and quantitative analyses of tube formation by HUVECs. Tube formation was eliminated in the COL-4A1-treated cells (with or without TNF-α). (**B**) COl-4A1 obviously increased the level of ROS in each group. (C) COl-4A1 increased the adhesive ability of each group. All images were captured at ×100 magnification. (*P < 0.05, **P < 0.01, ***P < 0.001).

Considering the correlation between COL-4A1 and the ability of HUVECs to form tubes, we further performed an in vitro experiment to simulate PE. COL-4A1 inhibited tube formation compared with that in the control group. Moreover, we added TNF-α to simulate PE, and the results were consistent (Fig. 3A). Similarly, we detected the levels of ROS and adhesive ability under both conditions (with or without TNF-α), and the results are depicted in Fig. 3B,C. The image clearly shows that COL-4A1 further elevated the levels of ROS and adhesive ability.

### The results of qRT-PCR and RNA sequencing

**Figure 4:**
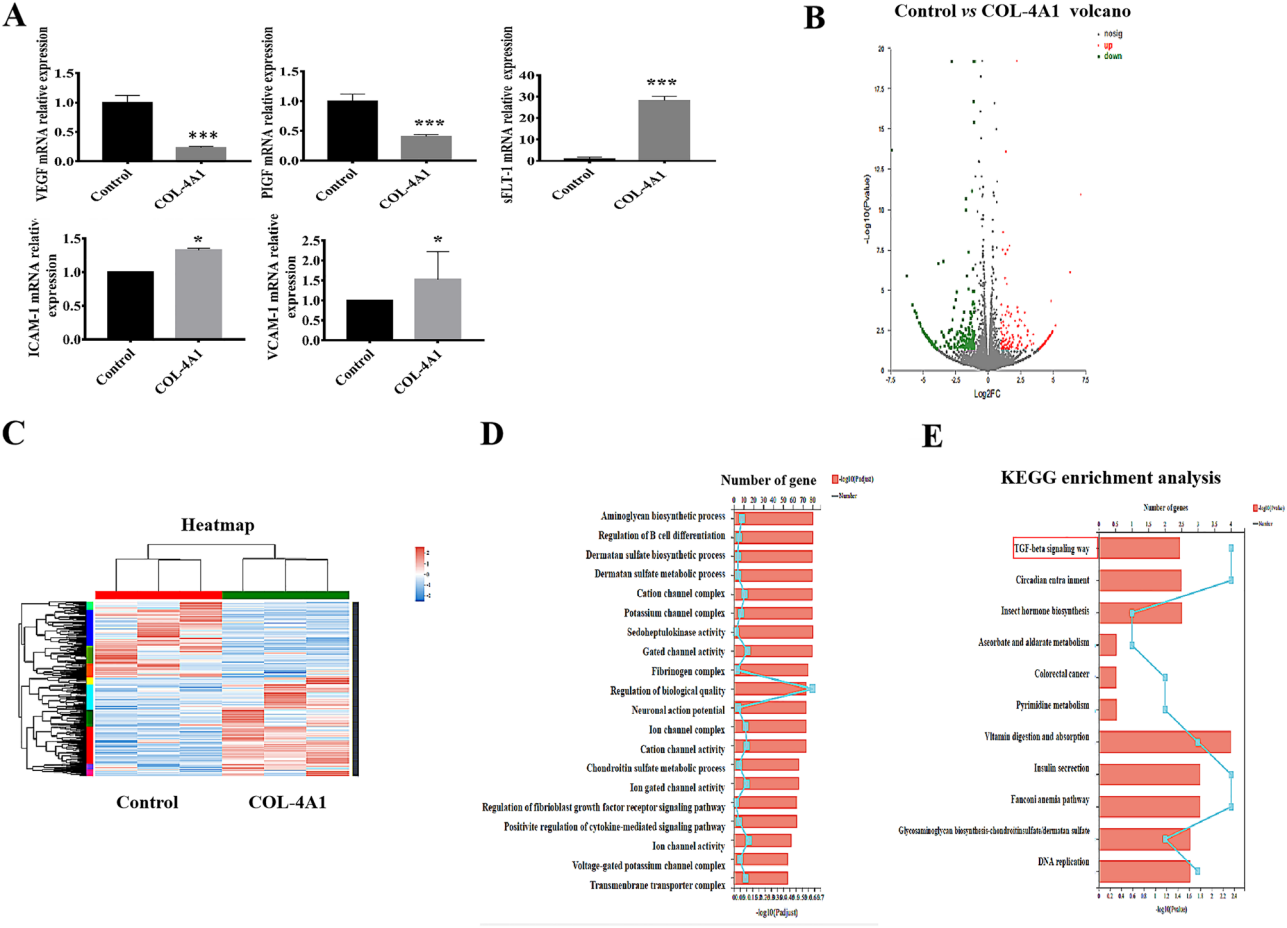
qRT-PCR assay and RNA sequencing of control *vs* COL-4A1-treated HUVECs. (**A**) Relative PIGF, VEGF, sFLT-1, ICAM-1 and VCAM-1 mRNA expression levels in each group. (**B**) Volcano plot. (**C**) Heat map. (**D**) Results of GO analysis. (**E**) Results of KEGG enrichment analysis. *PIGF* placenta growth factor, *VEGF* vascular endothelial growth factor, *sFLT-1* soluble fms-like tyrosine kinase-1, *ICAM-1* intercellular cell adhesion molecule-1, *VCAM-1* vascular cell adhesion molecule-1, *GO* Gene Otology, *KEGG* Kyoto Encyclopaedia of Genes and Genomes. (*P < 0.05, **P < 0.01, ***P < 0.001).

We detected VEGF, PIGF, sFLT-1, ICAM-1 and VCAM-1 mRNA expression, and the results are shown in Fig. 4A. The expression levels of VEGF and PIGF were decreased in the COL-4A1 group (*P* < 0.001). However, compared to those in the control group, sFLT-1 (*P* < 0.001), ICAM-1 (*P* < 0.05) and VCAM-1 (*P* < 0.05) mRNA expression levels were elevated in the COL-4A1 group. To further understand the mechanism of COL-4A1, transcriptome sequencing of RNA from HUVECs in the control and COL-4A1 group were performed. Heatmap revealed that differential genes which were detected with and without COL-4A1 treatment (*P* < 0.05, fold change = 2, Fig. 4C). Moreover, we further analyzed the differentially expressed genes through a volcano plot (Fig. 4B) and we found more down-regulated genes than up-regulated genes in the COL-4A1 *vs* control group (321 *vs* 209). To further understand the potential mechanism of COL-4A1, GO and KEGG analysis were conducted. GO analysis showed that the genes are mainly focused on the “regulation of biological quality” pathway (Fig. 4D). The TGF-β signalling pathway has been reported to inhibit HTR-8/SVneo cell and HUVECs proliferation, migration, invasion and tube formation^30^. KEGG analysis also showed that the TGF-β signalling pathway may be the pathway by which COL-4A1 functions (Fig. 4E).

### The results of pull-down assays and mass spectrometry assays

**Figure 5:**
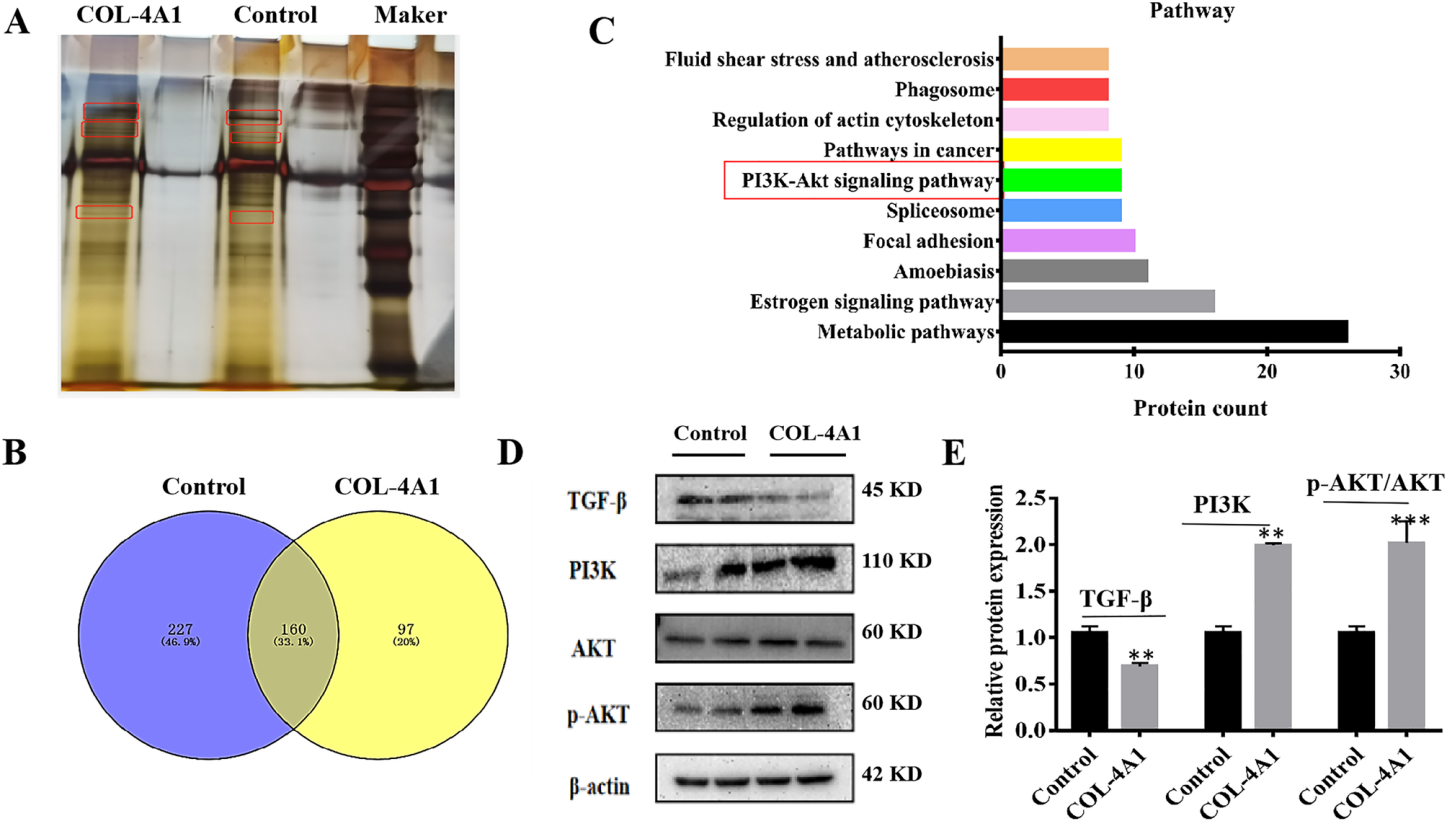
Differences in protein levels between the control and biotin COL-4A1 groups, as determined by pull-down assays, mass spectrometry and WB assays. (**A**) SDS-PAGE following pull-down assays with each group; the gels underwent silver staining. Differential protein fragments are marked with a red box. (**B**) Venn diagram. (**C**) Pathway analysis. (**D**, **E**) Western blotting (WB) analysis of antibodies (TGF-β, PI3K, p-AKT and AKT) in the control and COL-4A1 groups. (*P < 0.05, **P < 0.01, ***P < 0.001).

To further explore the pathway by which polypeptide COl-4A1 function, biotin COL-4A1 was synthesized for pull-down assays in which several differing protein fragments were obtained (Fig. 5A). Additionally, a Venn diagram of the mass spectrometry results indicated 97 proteins whose expression differed between the control and COL-4A1 groups (Fig. 5B). Furthermore, a potential protein polymerization pathway map was constructed (Fig. 5C). The classic PI3K-AKT pathway was included in this map (*P* < 0.05). Some reports have stated the pathway PI3K/AKT, which may contribute to the pathology of PE. For example, PI3K/AKT pathway plays a key role in cell metabolism and proliferation, inhibiting tube formation and mediating inflammatory processes^27,31^.

### The underlying mechanism by which COL-4A1 may function

Based on our results of RNA sequencing and mass spectrometry analysis, and we searched for the functions of the associated proteins with both GO and KEGG pathway analysis. Furthermore, the TGF-β signalling pathway was reported to inhibit HTR-8/SVneo cell and HUVECs proliferation, migration, invasion and tube formation ^30^. Additionally, some reviews have reported that PI3K/AKT, which may contribute to the pathology of PE, plays a key role in cell metabolism and proliferation, inhibiting tube formation and mediating inflammatory processes^27,31^. Hence, we hypothesized that COL-4A1 functions through the TGF-β/PI3K/AKT pathway. Then, we verified this hypothesis by pull-down and WB assays. Based on Fig. 5D,E, the protein expression of TGF-β was decreased (*P* < 0.01), but PI3K expression and the p-AKT/AKT ratio were increased (*P* < 0.05, *P* < 0.001).

## Discussion

In recent years, polypeptides have been a hot topic in research. Polypeptides have been applied in many fields as therapeutics and biomarkers and in the vaccine field^32^. A review published in 2020 suggested that polypeptides may be anti-infective drugs against different pathogens with potential biotechnological applications^33^. Studies on diabetes and polypeptides have reported that the effect of diabetes drugs and polypeptides in vivo is therapeutic, but the specific mechanism remains unclear; another study indicated that glucagon-like peptide-1 (GLP-1) stimulates insulin secretion, which plays a role in type 2 diabetes^34,35^. Moreover an in vitro model suggested that GLP-1 improves the function of endothelial nitric oxide synthase (eNOS) to prevent or delay the formation of atherosclerosis in diabetes mellitus; study in Human umbilical vascular endothelial cells (HUVECs) showed that GLP-1 promotes angiogenesis^36,37^. The polypeptide ELABELA has become a research focus in many fields (hypertension, CVD, renal disease, thyroid gland disease and PE) in recent years, and the latest progress has supported and highlighted its therapeutic potential^38,39^. There is still a lack of research on the peptide COL-4A1 and PE, thus the current study is focused on exploring the association between COL-4A1 and PE (Supplementary Information).

Shallow placental implantation and insufficient remodelling uterine spiral artery have been recognized as pathologies of PE for many years. Our results are consistent with this conclusion. From our above results, we know that in vivo, the model rats showed PE-like symptoms: increased blood pressure, FGR, and placenta and kidney abnormalities. In vitro, COL-4A1 could inhibit proliferation, migration and tube formation and caused an increase in ROS and adhesive capacity. A review published in Hypertension suggests that the pathology of PE includes inflammation and immunological factors, but oxidative stress and imbalance between angiogenic and antiangiogenic factors are also important pathological mechanisms that cause PE^40^. Compared to cells without COL-4A1 treatment, cells treated with COL-4A1 showed elevated VEGF mRNA expression and decreased PIGF expression through the results of qRT-PCR. Additionally, through ROS and adhesion assays, we discovered that cells treated with COL-4A1 had elevated ROS levels and an increased adhesive capacity. These results are consistent with the vascular endothelial damage theory^14^. When vascular endothelial cells are damaged, proinflammatory factors are expressed, angiogenic and antiangiogenic factors become unbalanced, ROS are produced, and the capacity to adhere is elevated.

The TGF-β and PI3K/AKT signalling pathways are common to PE and play key roles in many cellular processes, such as proliferation, migration, apoptosis, and angiogenesis^27,31,41,42^. In recent years, many researches on TGF-β has been carried out. TGF-β signalling has been reported to be dysregulated in early-onset severe PE^43^. The TGF-β signalling pathway includes the Smad‐dependent pathway and Smad‐independent pathway, which contains the PI3K/AKT pathway. Furthermore, studies have suggested that TGF-β/PI3K/AKT is related to cell migration^41,42^. Additionally, some reviews have reported that PI3K/AKT, which may contribute to the pathology of PE, plays key roles in cell metabolism, proliferation, the inhibition of tube formation and the mediation of inflammatory processes^27,31^. The results of a 2019 bioinformatics analysis suggested that the TGF-β signalling pathway inhibited HTR-8/SVneo cell and HUVECs proliferation, migration, invasion and tube formation^30^. However, the correlation between the TGF-β superfamily and PE is complex^30,44–48^. In 2006, a study suggested that PE impared TGF-β1 pathway, and decreased the amount of TGF-β1^45^. However, another study demonstrated that the levels of plasma TGF-β1 and TGF-β2 are elevated in PE^47^. Based on our RNA sequencing and mass spectrometry analysis results, we further performed WB assays. Based on the results of our WB assays, the protein expression of TGF-β was decreased (*P* < 0.01), but PI3K expression and the p-AKT/AKT ratio were increased. We considered that COL-4A1 may function through targeting the TGF-β pathway, decreasing TGF-β, activating the PI3K/AKT pathway and eventually causing PE.

The strengths of this study includes not only in vivo animal experiments but also in vitro cell experiments. Further this is the first study to study the correlation between polypeptide COL-4A1and PE. In summary, the present study highlights the correlation between COL-4A1 and PE, as the COL-4A1 peptide caused PE-like symptoms by inhibiting cell proliferation and migration. COL-4A1 may function through the TGF-β/PI3K/AKT pathway. Therefore, this research provides a basis for the pathogenesis of PE and provides a promising potential target for treatment.

## Supplementary Information

Supplementary Information.
