# Supplementary material for: The COL-4A1 polypeptide destroy endothelial cells through the TGF-β/PI3K/AKT pathway
Source: Sci Rep. 2021 Aug 3;11:15761. doi: 10.1038/s41598-021-94801-5 (PMC8333066; doi:10.1038/s41598-021-94801-5)
Supplement: Supplementary file 1 — Supplementary Information. [file 41598_2021_94801_MOESM1_ESM.pdf]

# The COL-4A1 polypeptide destroy endothelial cells through the TGF-β/PI3K/AKT pathway

Ting Li[1]†, Zhonghui Ling[1]†, Kaipeng Xie[1], Yixiao Wang[1], Zhijing Miao[1], Xiaohong Ji[1], Jingyun Li[1], Wenwen Hou[1], Qiuqin Tang[1], Xiaojie Yuan[1], Nan Li [1], Chanjuan Li[1]*, Hongjuan Ding[1]*

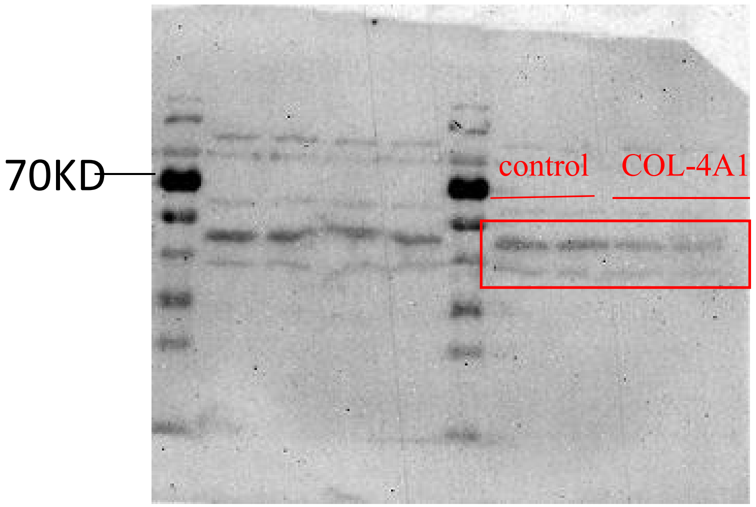


Fig. S1. The image of TGF-β in WB.

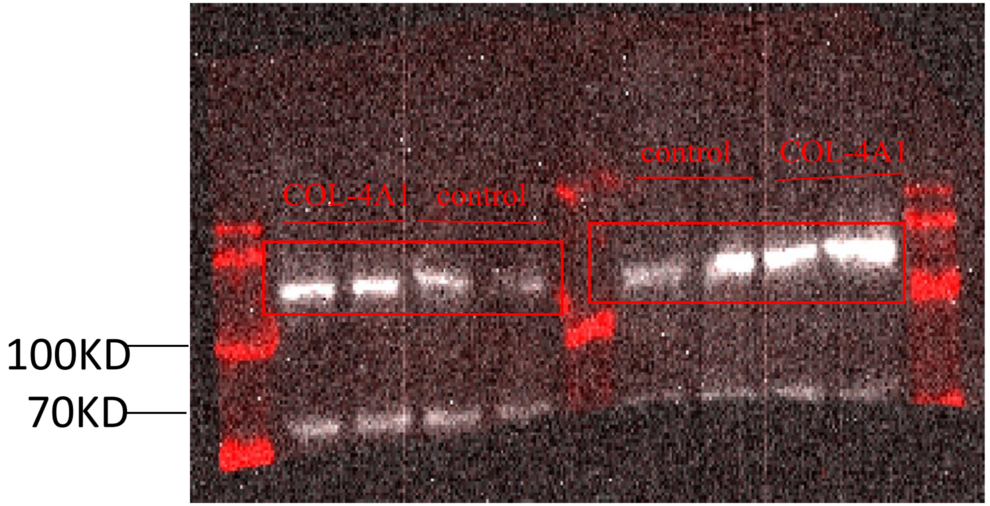


Fig. S2. The image of PI3K in WB.

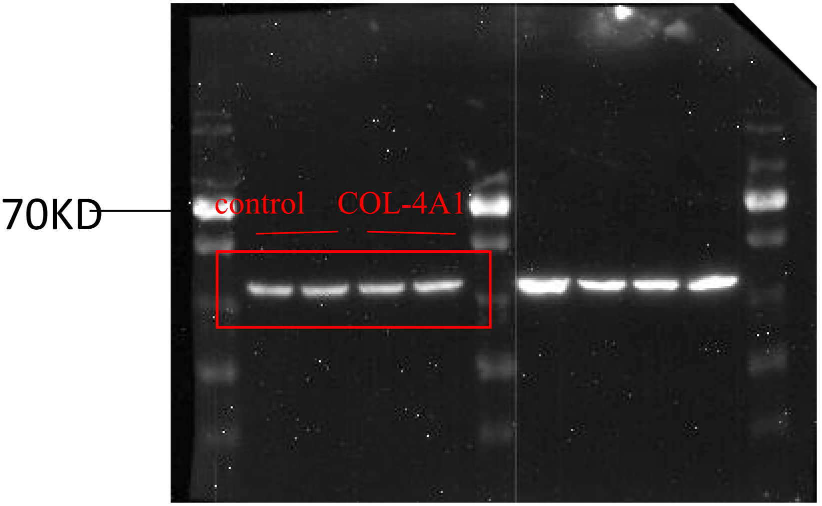


Fig. S3. The image of β-actin in WB.

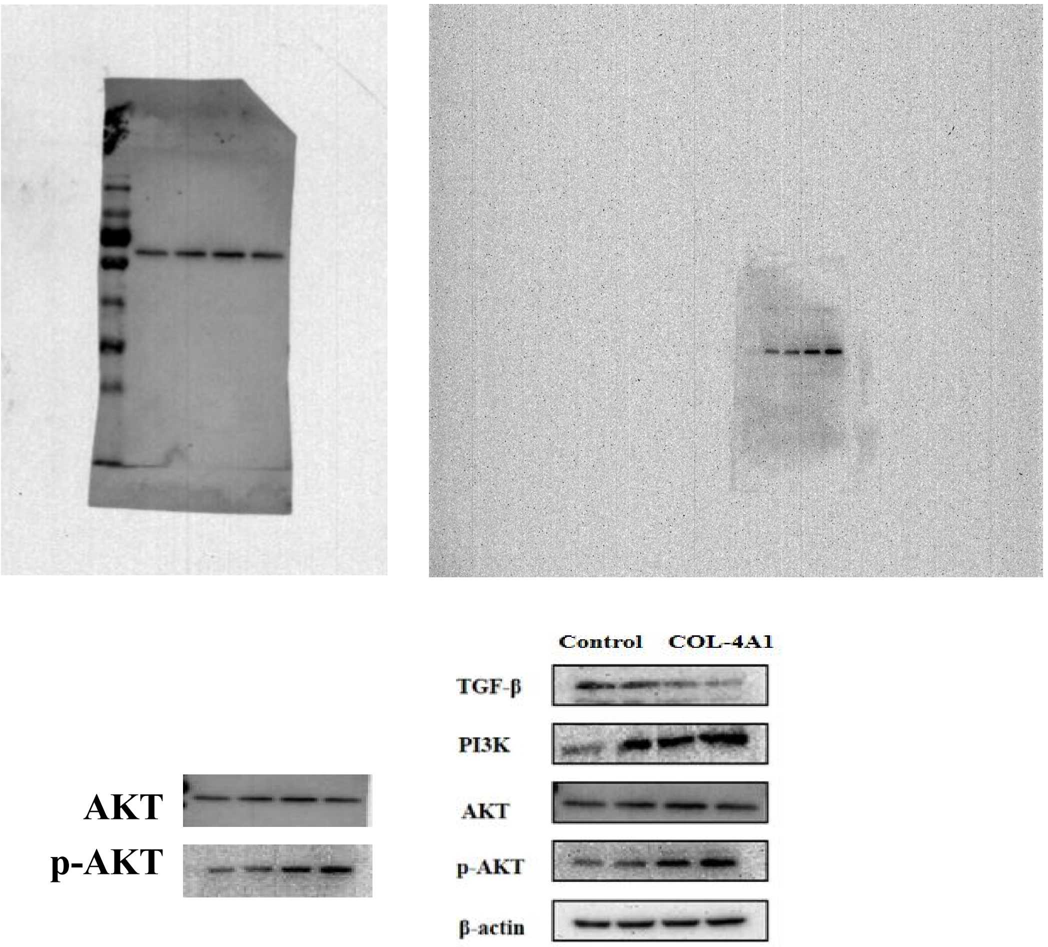


Fig S4. The image of AKT and p-AKT in WB.

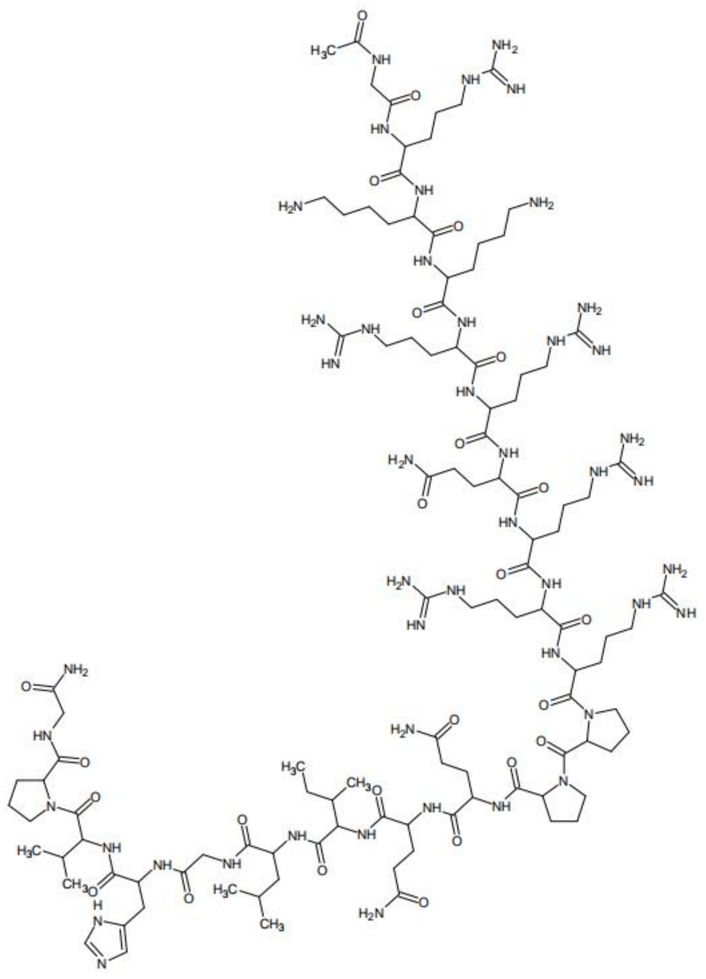

Fig S5. The structure of the polypeptide COL-4A1.

**Table 1.** Primer sequence for qRT-PCR

| Gene name | Forward primer (5' to 3') | Reverse primer (5' to 3') |
|---|---|---|
| VEGF | AGGTCCCCTTCTTCAGGAAACG | TCCAGGCTGTGCTCAGGAAAAG |
| PIGF | GAGACCCACAGACTGCCAC | ACCTTGGCCGGAAAGAACAA |
| sFlt-1 | TTTGCCTGAAATGGTGAGTAAGG | TGGTTTGCTTGAGCTGTGTTC |
| ICAM-1 | CAATGTGCTATTCAAACTGCCC | CAGCGTAGGGTAAGGTTCTTG |
| VCAM-1 | CAAAGGCAGAGTACGCAAAC | ACAGGATTTTCGGAGCAGG |
| GAPDH | GGAGCGAGATCCCTCCAAAAT | GGCTGTTGTCATACTTCTCATGG |

Abbreviation: VEGF, vascular endothelial growth factor; PIGF, placenta growth factor; sFlt-1, soluble fms-like tyrosine kinase-1; ICAM-1, intercellular cell adhesion molecule-1; VCAM-1, vascular cell adhesion molecule-1; GAPDH, glyceraldehyde-3phosphate dehydrogenase.

Figure legends:

Figure 1. COL-4A1 aggravated PE-like symptoms in a rat model treated with or without LPS. (A) The SBP of the control (saline) (n = 6), LPS (PE) (n = 6) and LPS+COL-4A1 (PE + COL-4A1) (n = 6) groups was evaluated on GD 4, 7, 11, 15, and 19. (B) Graph showing BP fluctuations in the model rats. (C, D) Images of the placenta and pup and their weights in each group. (E) Kidney and placenta sections from each group were subjected to HE staining. (F) Urinary albumin levels on GD 19. LPS, lipopolysaccharide; SBP, systolic blood pressure; GD, gestational day; PE, preeclampsia. (* $P < 0.05$, ** $P < 0.01$, *** $P < 0.001$).

Figure 2. The proliferation and migration of HUVECs were significantly inhibited by COL-4A1. (A) HUVECs were treated with or without COL-4A1 and with or without TNF-α before CCK-8 proliferation assays. (B, C, D, E) Wound-healing and Transwell assays were used to detect the ability of in the cells in each group to migrate. All images were captured at 200× magnification. (* $P < 0.05$, ** $P < 0.01$, *** $P < 0.001$).

Figure 3. Effects of COL-4A1 on tube formation, ROS levels, and adhesive ability. (A) Qualitative and quantitative analyses of tube formation by HUVECs. Tube formation was eliminated in the COL-4A1-treated cells (with or without TNF-α). (B) COl-4A1 obviously increased the level of ROS in each group. (C) COl-4A1 obviously increased the adhesive ability of each group. All images were captured at ×100 magnification. (* $P < 0.05$, ** $P < 0.01$, *** $P < 0.001$).

Figure 4. qRT-PCR assay and RNA sequencing of control vs COL-4A1-treated HUVECs. (A) Relative PIGF, VEGF, sFLT-1, ICAM-1 and VCAM-1 mRNA expression levels in each group. (B) Volcano plot. (C) Heat map. (D) Results of GO analysis. (E) Results of KEGG enrichment analysis. PIGF, placenta growth factor; VEGF, vascular endothelial growth factor; sFlt-1, soluble fms-like tyrosine kinase-1; ICAM-1, intercellular cell adhesion molecule-1; VCAM-1, vascular cell adhesion molecule-1; GO, Gene Otology; KEGG, Kyoto Encyclopaedia of Genes and Genomes. (* $P < 0.05$, ** $P < 0.01$, *** $P < 0.001$).

the control and COL-4A1 groups. (* $P < 0.05$, ** $P < 0.01$, *** $P < 0.001$).

Figure 5. Differences in protein levels between the control and biotin COL-4A1 groups, as determined by pull-down assays, mass spectrometry and WB assays. (A) SDS-PAGE following pull-down assays with each group; the gels underwent silver staining. Differential protein fragments are marked with a red box. (B) Venn diagram. (C) Pathway analysis. (D, E) Western blotting (WB) analysis of antibodies (TGF-β, PI3K, p-AKT and AKT) in
